# Express detection of visual objects by primate superior colliculus neurons

**DOI:** 10.1038/s41598-023-48979-5

**Published:** 2023-12-08

**Authors:** Amarender R. Bogadhi, Ziad M. Hafed

**Affiliations:** 1 Werner Reichardt Centre for Integrative Neuroscience, University of Tübingen, Otfried-Müller Str. 25, 72076 Tübingen, Germany; 2 Hertie Institute for Clinical Brain Research, University of Tübingen, 72076 Tübingen, Germany; 3 Central Nervous System Diseases Research, Boehringer Ingelheim Pharma GmbH & Co. KG, 88400 Biberach, Germany

**Keywords:** Sensory processing, Object vision, Superior colliculus

## Abstract

Primate superior colliculus (SC) neurons exhibit visual feature tuning properties and are implicated in a subcortical network hypothesized to mediate fast threat and/or conspecific detection. However, the mechanisms through which SC neurons contribute to peripheral object detection, for supporting rapid orienting responses, remain unclear. Here we explored whether, and how quickly, SC neurons detect real-life object stimuli. We presented experimentally-controlled gray-scale images of seven different object categories, and their corresponding luminance- and spectral-matched image controls, within the extrafoveal response fields of SC neurons. We found that all of our functionally-identified SC neuron types preferentially detected real-life objects even in their very first stimulus-evoked visual bursts. Intriguingly, even visually-responsive motor-related neurons exhibited such robust early object detection. We further identified spatial frequency information in visual images as an important, but not exhaustive, source for the earliest (within 100 ms) but not for the late (after 100 ms) component of object detection by SC neurons. Our results demonstrate rapid and robust detection of extrafoveal visual objects by the SC. Besides supporting recent evidence that even SC saccade-related motor bursts can preferentially represent visual objects, these results reveal a plausible mechanism through which rapid orienting responses to extrafoveal visual objects can be mediated.

## Introduction

Object detection and recognition are fundamental components of primate vision, and a substantial number of visual cortical areas are dedicated to processing visual objects^1–5^. However, vision does not occur in complete isolation of behavior, and an element of visual object processing in the brain must facilitate active orienting in association with objects, whether to avoid threats^6^ or to foveate and further process behaviorally-relevant items. Indeed, certain classes of visual objects, like faces, easily pop out from visual scenes with very short latencies^7^, and short-latency eye movements can likewise be automatically captured by completely task-irrelevant object images^8^.

The speed with which orienting phenomena associated with visual object recognition proceed points to the presence of subcortical mechanisms for visual object processing. Indeed, in 1974, Updyke^9^ observed neurons in the superior colliculus (SC), a site of convergence for retinal and extra-retinal visual signals^10^, that were particularly sensitive to three-dimensional objects, and SC cells sensitive to complex visual stimuli were also reported by Rizzolatti and colleagues in 1980^11^. More recently, a series of seminal studies explored the roles of the SC and pulvinar in the processing of face and snake images^12–18^. These studies concluded that the SC may be part of a fast detection network for visual threats and ecologically-relevant faces, which can in turn influence emotions^6^. Consistent with this idea, recent work confirmed the presence of rapid preference for face images in SC neural responses^19^.

Because the SC is also shown to contribute to a variety of important cognitive processes like target selection, visual attention, and perceptual decision making^20–26^, and given that SC activity can influence cortical areas through different thalamic circuits^27–30^, it stands to reason that the SC may be involved in object processing in a more general way than being specifically tuned for processing snakes and faces. In fact, experimental manipulation of SC activity is associated with altered object selectivity in a patch of the ventral visual processing stream of the cortex^31^, and, similarly, the SC has a dedicated primary cortical area in mice^32^.

Importantly, the SC possesses a kind of privileged access to the saccadic system’s motor periphery, and even saccade-related SC motor bursts embed within them a preferential visual-object representation^33^. Therefore, a generalized detection of extrafoveal visual objects by the SC not only can support rapid orienting behaviors, which are facilitated by visual objects^8^, but it can also help bridge visual object representations across eye movements^33^. As a result, there is a pressing need to investigate whether, and how, neurons in the primate SC detect visual objects in the extrafoveal visual space.

In this study, we investigated this topic by presenting seven different visual object images to individual SC neurons with extrafoveal response fields, along with various control versions of the same images disrupting object information. We found rapid and sustained detection of the extrafoveal visual objects by all visually-responsive SC neuron types. We also observed that SC tuning to spatial frequency information in images^34,35^ can facilitate the fastest components of SC visual object processing. These results, demonstrating that generalized visual object detection is a robust property of the primate SC, are intuitive in hindsight, especially because detecting objects outside of the fovea is very often a necessary precursor to foveating objects for a detailed visual analysis in normal active visual behavior.

## Results

**Figure 1:**
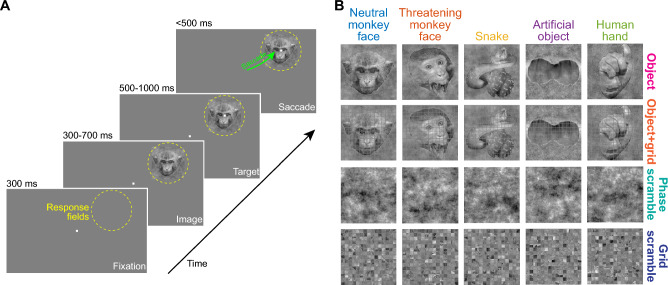
Behavioral task and example images. (**A**) Each task started with a central fixation spot. At the approximate response field (RF) locations of the recorded neurons in a given session (yellow dashed circle), an image appeared during fixation. After 300–700 ms from image onset, a saccade target appeared on top of the image for another fixation interval (500–1000 ms). The fixation spot then disappeared, instructing the monkey to generate a saccade towards the target on top of the image (green arrow). Our focus here is on the image onset component of the task; effects in the saccade component were documented recently^33^. (**B**) Example images from a given session. The fruit image from the session is shown in Fig. 3, and the human neutral face image is not shown for data privacy reasons. The top row shows the real object images, and the second row shows these images with the grid overlay. The third row shows the phase-scrambled images, and the bottom row shows the grid-scrambled images (“Methods”).

To investigate visual object detection by SC neurons, we employed a version of the delayed visually-guided saccade task with the saccade target overlayed on images in the response fields (RF’s) of the recorded SC neurons (Fig. 1A; “Methods”). In every experimental session, we used a novel set of 28 different images, drawn from seven different object categories and their corresponding control images (Fig. 1B; “Methods”). The control images were luminance- and spectrum-matched non-object images (“Methods”): phase-scrambled controls had the same spatial frequency content as the real object images, but with spectral phase scrambling; grid-scrambled images had small, square patches (grids) containing identical copies of small patches from the original images, but with randomized locations (Fig. 1B). The grid-scrambled images maintained local image properties but disrupted global form information, and we explicitly tested the case of peripheral objects filling RF’s (and with local features smaller than these RF’s; “Methods”). Finally, since grid scrambling also introduced a square grid of hard edges between the scrambled image patches (altering the spatial frequency content of the images), we also checked whether object detection by SC neurons was significantly disrupted by overlaid grids presented over the intact objects (object + grid images; Fig. 1B). Thus, each neuron was tested with seven different object images (of seven different categories) and four different image types: two being coherent objects (object and object + grid) and two being image-matched, non-object images (grid-scrambled and phase-scrambled).

### The very first SC visual responses differentiate between object and non-object stimuli

**Figure 2:**
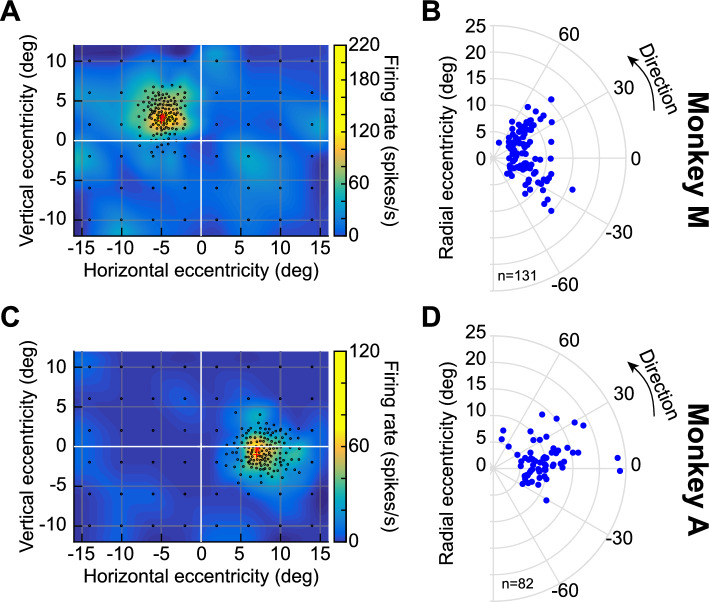
Response field (RF) locations of the recorded neurons. (**A**) Visual RF of an example neuron recorded from monkey M. Each black circle indicates a sampled location in which we presented a small white spot during fixation. The pseudocolor surface indicates the mean firing rate emitted by the neuron in a visual epoch 40–140 ms after spot onset (we interpolated across space between the sampled locations to obtain the pseudocolor surface). The neuron’s RF occupied the upper left quadrant, and our online estimate of its hotspot is indicated by the red asterisk. The red cross indicates where we placed the image during the main experiment. (**B**) All RF hotspot locations from monkey M (remapped to one hemifield for easier viewing). Our neurons were extrafoveal. (**C**) Visual RF of an example neuron recorded from monkey A. The same conventions as in (**A**) apply. The neuron occupied the lower right quadrant. (**D**) All RF hotspot locations from monkey A, showing similar coverage to monkey M. For purely motor neurons, RF hotspot locations in (**B**,**D**) were obtained from the saccade-related, rather than visual, responses.

We recorded SC neural responses to images presented in their RF’s. The RF’s of the neurons were determined independently using the classic delayed visually-guided saccade with a small white spot (“Methods”); the center and extent of each RF were estimated online (Fig. 2A,C), to determine the position and size of the image patch. We also confirmed that the RF’s of all of the recorded neurons were away from the central 2° of the visual field in both monkeys (Fig. 2B,D), with eccentricities spanning up to 24°.

**Figure 3:**
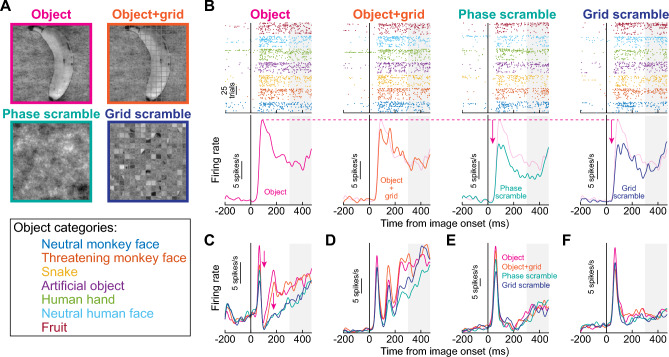
Early and sustained enhancement of superior colliculus (SC) visual activity for real-life object images. (**A**) We presented an image of real-life objects (e.g. banana) as well as multiple variants of it. The object + grid image overlaid a grid creating small square patches of image regions. The phase-scrambled image contained the same spatial frequency content as the object image, but with scrambled phase information. And, the grid-scrambled image had randomized grid locations from the object + grid image. In total, we tested seven different object images, from seven categories, spanning faces, animals, and artificial objects. See also Fig. 1. (**B**) Each column shows the responses of an example neuron under the four different image conditions. The leftmost column shows responses to intact object images. Top: individual trial spike time rasters showing responses to each object category (according to the color legend of object categories in **A**); bottom: average firing rate plot pooling the seven different object images together (we did not assess object preference in this study because we only had one exemplar per object category within each session). The neuron exhibited a robust visual burst followed by sustained activity. In the second column, the overlaid grid minimally altered the response (the faint curve is a copy of that in the leftmost column for reference). However, both the phase-scrambled (third column) and grid-scrambled (fourth column) conditions were associated with significantly weaker activity. (**C**–**F**) Four additional example neurons showing similar results. The object and object + grid conditions always had the highest initial visual bursts. Moreover, sustained activity was higher for the object and object + grid conditions than for the scrambled conditions. The gray shaded regions in (**B–F**) denote the time at which the saccade target could appear in the subsequent stages of the trials (Fig. 1).

We analyzed SC visual responses to images of real-life objects appearing within the recorded neurons’ RF’s. Initial and sustained SC visual responses were systematically higher for real object stimuli than for non-object images. Consider the example neuron of Fig. 3B. In both the object and object + grid conditions (leftmost two columns), the neuron’s visual response was higher than in the phase- and grid-scrambled conditions (rightmost two columns). Therefore, the neuron discriminated between intact object and non-object stimuli even within its very first, initial visual burst (i.e. within approximately 50 ms from image onset).

In Fig. 3C–F, we also show results from four additional example neurons. In all cases, the initial stimulus-driven visual bursts were the highest for real object images and/or object + grid images. Moreover, sustained visual activity was clearly higher for the object and object + grid images than for the phase- and grid-scrambled images, and this was the case even for the neurons with relatively low sustained activity (Fig. 3E,F). Note that in these analyses, we pooled all seven object images of the session together after confirming that there was no consistent population preference among the 7 categories of images that included faces (“Methods”). Also note that starting at 300 ms after image onset (gray shaded regions), the saccade target could appear for the next stages of the behavioral task (“Methods”). Therefore, in all subsequent analyses, we focused only on the first 300 ms of neural responses; saccade-related effects were detailed elsewhere^33^. In all, the five example neurons of Fig. 3 suggest the presence of both very early as well as sustained discrimination of object and non-object images by primate SC neurons. Next, we proceeded to quantify this discrimination of object and non-object images in our SC neural population.

We confirmed that, across the population, early SC visual bursts robustly discriminated between object and non-object images. We did so by assessing the discriminability of firing rates between the object and grid-scrambled conditions; we performed a running receiver operating characteristic (ROC) analysis on the neural responses, using 40 ms time bins in steps of 10 ms (“Methods”). For each time bin around image onset, we collected firing rates from each condition (either intact object or grid-scrambled image) pooled for all seven object categories, and we then calculated the area under the ROC curve (AUC) between the two distributions. AUC values significantly different from 0.5 indicated discriminable firing rate distributions between object and grid-scrambled images (“Methods”).

**Figure 4:**
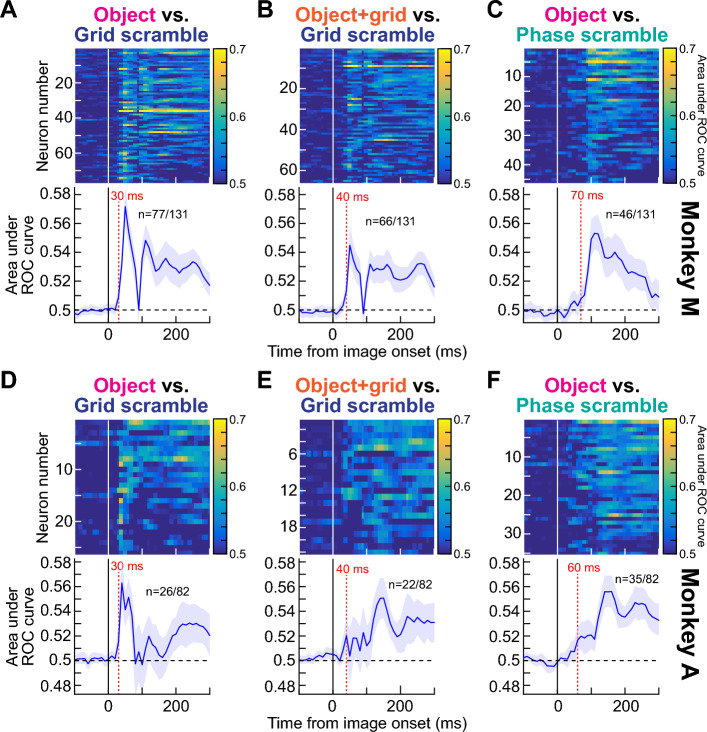
Early and late detection of visual objects by SC neurons. (**A**) For each neuron in monkey M, we compared distributions of firing rates (in 40 ms time bins) between intact and grid-scrambled object images using ROC analyses (“Methods”). For each neuron with a significant AUC (area under ROC curve) value in the interval 0–300 ms from image onset (n = 77), we plotted AUC as a function of time in the top panel (the color indicates the AUC value). The bottom panel plots the average of all neurons’ AUC time courses (error bars denote 95% confidence intervals across the population), showing an initial robust peak followed by sustained elevation. The dashed vertical line marks the first time point after stimulus onset for which the AUC value of the population was significantly deviated away from 0.5 (30 ms). (**B**) Same analysis but comparing object + grid images to grid-scrambled images. The overlay of a grid on top of the images (Figs. 1B, 3A) was not enough to strongly alter the ability of the neurons to detect visual objects, but the altered spatial frequency content of object + grid images slightly modified the early (< 100 ms) AUC values (see **C**). (**C**) Same analysis but comparing object images to phase-scrambled images. Here, the early peak in AUC discrimination performance was attenuated, suggesting that the spatial frequency content of object images contributes to early object detection mechanisms by the SC. (**D**–**F**) Same as (**A**–**C**) but for monkey A. The results in both animals were consistent with each other. Figure 5 shows related analyses controlling for the effects of microsaccades.

In each monkey, we accepted a neuron as significantly detecting objects versus non-object stimuli if it had a significant AUC value in any time bin within 0–300 ms from image onset (“Methods”). We used a broad time window (0–300 ms) to avoid selection bias towards neurons with early or late discrimination. Out of 131 neurons in monkey M (including task-irrelevant ones like purely motor neurons), 77 showed significant discrimination performance for intact objects relative to grid-scrambled images. In monkey A, 26 out of 82 total neurons (again including task-irrelevant ones like purely motor neurons) did so. Most importantly, in both monkeys, the highest discrimination performance always occurred in the very initial visual burst interval. This is illustrated in Fig. 4A for monkey M and Fig. 4D for monkey A (error bars denote 95% confidence intervals). Therefore, SC neurons detect extrafoveal visual objects in an express manner, consistent with behavioral evidence of an automatic influence of peripheral visual forms on target selection for eye movements^8^, and also consistent with results demonstrating altered cortical object selectivity with altered SC activity^31^.

Since grid scrambling necessarily entailed adding hard vertical and horizontal edges around each grid (see the example grid-scrambled image in Figs. 1, 3A), we also checked whether the results of Fig. 4A,D were trivially explained by these added edges. We, therefore, repeated the ROC analyses, but this time comparing the grid + object images to the grid-scrambled ones. Now, both image types had the same hard edges embedded within them, but the grid + object images preserved much of the form information in the original intact object images; the grid + object stimuli were akin to the objects being occluded by a thin rectangular mesh and thus still recognizable as coherent objects. We still found early and sustained discrimination performance in both monkeys (Fig. 4B,E), albeit, with weaker early discrimination in monkey A. Thus, the results of Fig. 4A,D were not explained by the slightly altered spatial frequency content introduced by the grids in the grid-scrambled images. We next explored spatial frequency effects more closely.

### The earliest phase of visual-object detection by SC neurons uses spatial frequency image content

**Figure 5:**
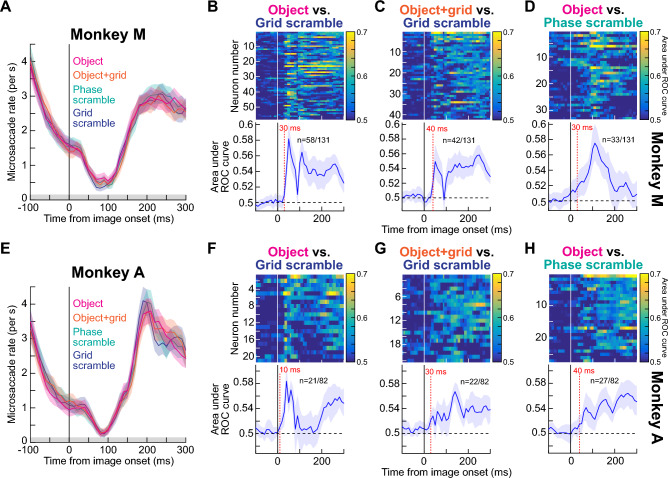
Discrimination between object and non-object stimuli by SC neurons’ visual responses, after controlling for microsaccades. (**A**) Microsaccade rate around the time of image onset from monkey M. A classic modulation of eye movement rate was present^41,42,48,49^. Note that microsaccade rate was negligible in the early visual burst interval of neural responses, due to the known phenomenon of microsaccadic inhibition. The relatively high (but declining) microsaccade rate before image onset was due to the short initial fixation interval of the task (Fig. 1), and therefore had some refixation saccades as the monkey was starting a new trial after the end of the previous one. The gray bar on the x-axis denotes the interval chosen for removing microsaccades in the control analyses of (**B**–**D**). (**B**–**D**) Same results as in Fig. 4A–C but after including only trials in which there were no microsaccades in the entire shown interval in (**A**) (− 100 ms to + 300 ms from image onset). The same qualitative results as in the main text were obtained. In fact, the AUC values here were generally higher than with all trials included, and significant AUC values now appeared as early in (**D**) as they did in (**B**, **C**). This is expected because microsaccades jitter images, and are associated with various effects on SC neurons’ firing rates^45–47,50^. (**E**) Same as (**A**) but for monkey A. (**F**–**H**) Same as (**B**–**D**) but for monkey A.

Because spatial frequency is expectedly relevant for visual object recognition^36–40^, and because primate SC neurons exhibit spatial frequency tuning^34,35^, we next asked how object detection performance as in Figs. 3, 4A,B,D,E depended on spatial frequency. We repeated the ROC analyses, but we now pitted intact object images against phase-scrambled images (“Methods”). In these latter images, there was no grid overlay, but the phases of the different spatial frequency bands of the images were randomized relative to the intact object image condition. We still found a substantial number of neurons in each monkey with significant AUC values in the first 300 ms after image onset (Fig. 4C,F), satisfying our criteria for object detection by SC neurons. Interestingly, the very earliest phase (< 100 ms) of AUC discrimination performance between intact and phase-scrambled images was significantly weaker than in the case of grid scrambling (Fig. 4A,D vs Fig. 4C,F). For example, across the population of significant neurons in each monkey in the phase-scrambled condition (46 in monkey M and 35 in monkey A), the average population AUC value first moved significantly away from 0.5 (at the 95% confidence level) at 70 ms and 60 ms after image onset for monkeys M and A, respectively (Fig. 4B,E). This contrasts with the earlier detection of objects with respect to grid-scrambled images (30 ms; Fig. 4A,D). This observation implies that in the very early phases (< 100 ms) of visual responses in our population, neural activity for the intact objects was more similar to that for phase-scrambled objects than it was to grid-scrambled images. We emphasize, however, that there was still significant, albeit slightly delayed, AUC discrimination performance between objects and phase-scrambled images within the initial visual burst phases of SC responses, and this is consistent with the five example neurons of Fig. 3 (also see Fig. 5D, H for clearer evidence of this across the population). Therefore, object detection by SC neurons in the very early phases (< 100 ms) of neural responses is partially, but not fully, mediated by the spatial frequency image processing capabilities of these neurons. This highlights an interesting potential functional role for spatial frequency tuning in primate SC neurons^34,35^, and it is consistent with an important role for spatial frequency information in object recognition^36–40^.

In longer intervals after image onset (i.e. > 100 ms), there was still significant AUC discrimination performance between the intact and phase-scrambled object images. This is clearly seen in Fig. 4C,F, in which significant AUC discrimination performance persisted at least until the next phase of the trials (> 300 ms). Such sustained effect might suggest a reverberation of object representation between the SC and other visual cortical areas associated with object recognition. For example, because object recognition may preferentially benefit from mid-spatial-frequency information^37–40^ and the SC is primarily low-spatial-frequency tuned^34^, feedback to the SC after the initial visual bursts can help to stabilize the SC representation for the detected objects for prolonged intervals. Therefore, object detection by SC neurons proceeds with both an early (< 100 ms) and a sustained (> 100 ms) phase; the early phase (< 100 ms) is supported by spatial frequency information that is intrinsically present in the SC neurons (Fig. 4A,D), and the later phase (> 100 ms) may use additional form information that could potentially be relayed to the SC from other brain areas (Fig. 4C,F).

We also analyzed microsaccades to remove potential eye movement confounds from our analyses. Microsaccade rate exhibited expected modulations as a function of time from image onset (Fig. 5A,E)^41–44^. This meant that in the early visual burst intervals of neural responses, there were already rare microsaccades due to microsaccadic inhibition. This ruled out a potential role for microsaccades in at least explaining the early visual burst interval results so far. However, we still repeated all analyses when excluding all trials containing microsaccades in the interval between -100 ms and + 300 ms from image onset. Our results were largely unchanged (Fig. 5B–D,F–H). In fact, the AUC discrimination performance improved slightly across the board (compare Fig. 5B–D,F–H to Fig. 4), as might be expected given that microsaccades can modulate SC visual bursts^45,46^, and also given that these movements can cause measurable visual reafferent SC neural modulations after image jitter^47^, both in a spatial-frequency dependent manner.

### Even visual-motor SC neurons detect objects in their very first visual responses

To further appreciate the SC’s role in express object detection, even within the initial visual bursts, we also considered this structure’s different functional neuron types. For example, it is well known that deeper-layer visual-motor neurons are relevant for a variety of cognitive processes like target selection, attention, and decision making^20–22,24–26,51^, in addition to their roles in eye movement generation^52–55^. So, we functionally classified our neurons according to classic visual and saccade-related response criteria (“Methods”), and we then explored object detection performance once again.

In both monkeys, most of our neurons were visual-motor-prelude neurons (“Methods”): they emitted visual bursts after stimulus onset, saccade-related bursts at saccade onset, as well as significant prelude activity (above baseline spiking rate) before saccade onset. We also encountered visual-motor neurons, which did not exhibit substantial delay-period (prelude) activity but were otherwise similar to visual-motor-prelude neurons. Finally, our database included a fewer number of purely visual neurons, which came in two primary flavors: visual neurons emitting a burst shortly after stimulus onset, and visual-delay neurons also exhibiting delay-period activity after the bursts.

**Figure 6:**
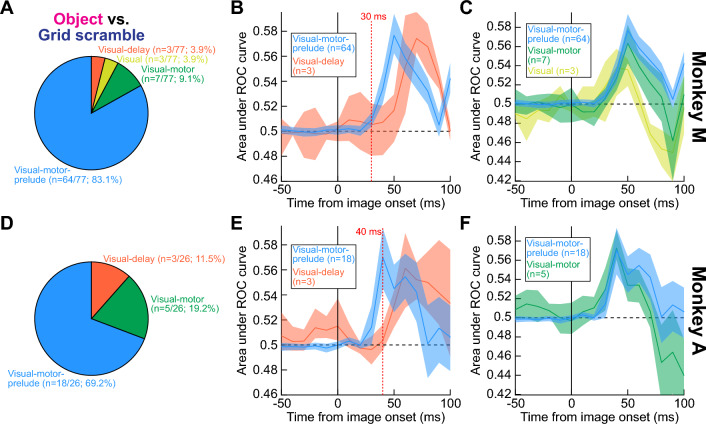
Express object detection even by visual-motor neurons. (**A**) Distribution of neuron types (“Methods”) exhibiting significant object detection performance in the data of Fig. 4A (monkey M). Visual-motor and purely visual neurons were both present. (**B**) When we compared the initial AUC discrimination performance between visual-motor-prelude and visual-delay neurons, we found earlier object detection by the visual-motor-prelude neurons (with the caveat of significantly fewer visual-delay neurons in the database). Error bars denote 95% confidence intervals. The dotted red vertical line provides an additional time reference for comparison. (**C**) Similarly, visual-motor neurons (without prelude activity) also exhibited early detection performance. In this panel, the curve from visual-motor-prelude neurons is replicated from (**B**) to facilitate comparison. In this animal, a few visual neurons were also encountered that exhibited object detection performance, and their results are shown in yellow. Thus, all visual and visual-motor neuron types detected objects in this animal, and it is interesting that even visual-motor neurons exhibited early detection. (**D**–**F**) Highly similar results from monkey A (again the red vertical line in **E** provides an additional time reference). Note that in this monkey, we did not encounter visual neurons, so they are not shown in (**F**) as they were shown in (**C**). Figure 7 provides further analyses of neuron types, focusing on later, sustained intervals of neural discharge.

All neuron types that we encountered exhibited significant object detection capabilities, and very similarly in both monkeys. For example, Fig. 6A,D shows the distribution of neuron types contributing to the results of Fig. 4A,D. Both visual-motor types were most frequent in both monkeys (likely due to the recording technique with thick electrode shanks; “Methods”), but purely visual neurons were also clearly present. Most interestingly, visual-motor neurons detected visual objects even earlier than visual-delay neurons in both monkeys (with the caveat that the number of the visual-delay neurons was relatively low). This result is illustrated in Fig. 6B,E: in both animals, visual-motor-prelude neurons exhibited high AUC discrimination performance (relative to grid-scrambled images) in their very initial visual bursts, and this high discrimination performance actually preceded the discrimination performance of visual-delay neurons. Even though the numbers of visual-delay neurons were relatively low in each animal, the effects in both animals were virtually identical, increasing our confidence in concluding that there is indeed very early object detection by visual-motor-prelude neurons. Such neurons detect visual objects as early as (if not earlier) than purely visual neurons (Fig. 6B,E; also see Fig. 6C,F).

We also found that prelude activity was not a prerequisite for visual-motor neurons to exhibit rapid object detection. Specifically, in Fig. 6C,F, we repeated the ROC analyses but now for the visual-motor neurons (green), which did not have substantial delay-period activity. For comparison, we also plotted the visual-motor-prelude neuron results from Fig. 6B,E again, to facilitate comparing the curves. Both neuron types exhibited similar early detection of intact visual objects relative to grid-scrambled images (similar results were also obtained with phase scrambling). In monkey M, we also had some purely visual (burst) neurons, and they also exhibited early object detection (yellow in Fig. 6C). Therefore, all the above results suggest that visual-motor SC neurons are a substantial contributor to the SC’s ability to rapidly detect visual objects.

**Figure 7:**
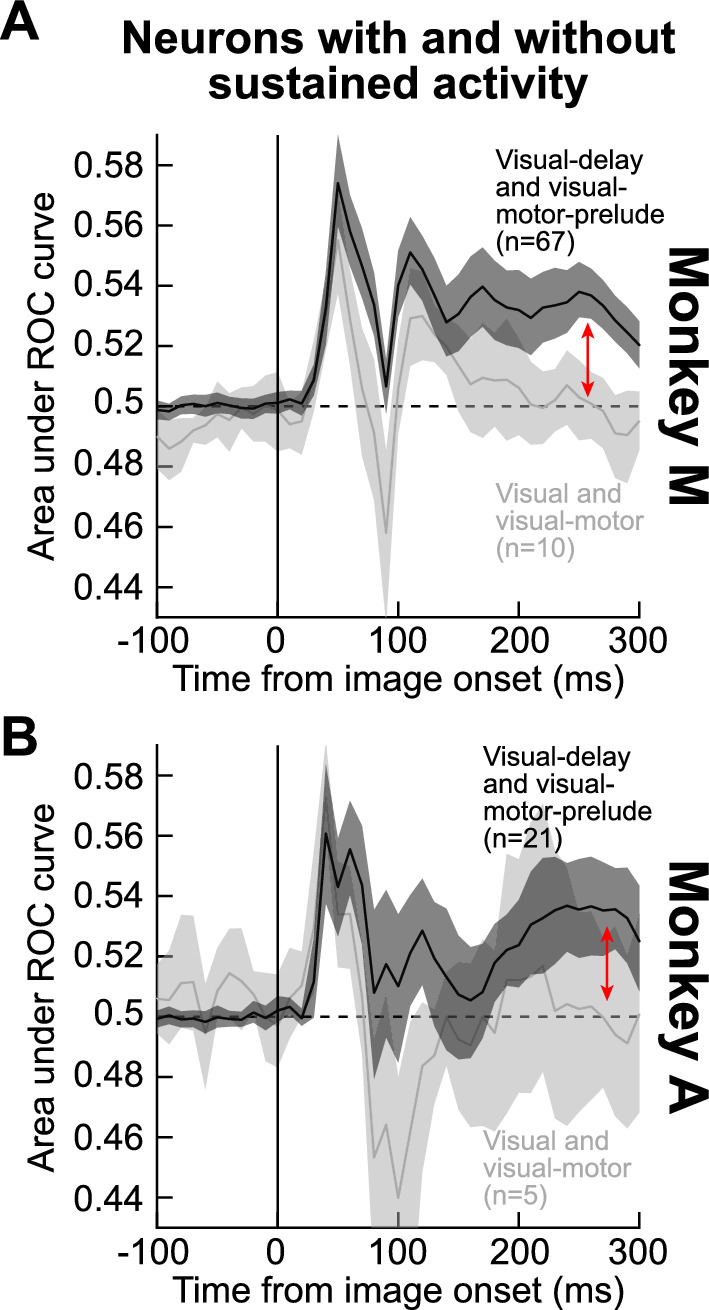
Neurons with sustained delay-period (prelude) activity allow sustained discrimination between object and non-object images in SC RF’s. (**A**) We performed our ROC analyses on object versus grid-scrambled images as in Figs. 4, 6, but this time by pooling only neurons with delay-period activity (visual-delay and visual-motor-prelude neurons) or neurons without (visual and visual-motor neurons). In the latter group, discrimination performance returned to baseline (light gray), whereas it remained significant throughout the sustained interval for the first group of neurons (see red vertical arrow). Error bars denote 95% confidence interval. (**B**) We observed very similar results in monkey A, although the smaller number of visual and visual-motor neurons (light gray) reduces the statistical confidence around this latter group of neurons.

Perhaps expectedly, the neurons that had sustained activity also showed sustained significant AUC discrimination performance between object and scramble images. For example, when we repeated the ROC analyses of Figs. 4, 6 for visual-motor-prelude and visual-delay neurons combined (both of which had sustained activity), and we compared them to visual-motor and visual neurons (both not having sustained activity), we found that the later (> 100 ms) AUC discrimination performance was systematically higher for the former group of neurons (Fig. 7). This observation suggests that sustained activity provides a plausible spiking substrate for encoding information about the visual images.

Therefore, not only do SC neurons detect extrafoveal visual objects early (Figs. 3, 4, 5), but they do so even if they are motor-related neurons (Fig. 6). Moreover, delay-period activity contributes to maintaining information about the intact object images for sustained intervals, as might be the case in a variety of cognitive tasks.

## Discussion

We found that all of our classified visual and visual-motor SC neuron types contributed to rapid detection of peripheral visual object images, with even deeper visual-motor neurons doing so in their very first visual bursts. Such visual-motor neurons are typically implicated in a variety of cognitive functions beyond saccade generation^20–22,56–58^, suggesting that the visual form information that they carry can influence such functions as well. Moreover, because of the intrinsic motor nature of these neurons, it would be very intriguing to think more deeply of the role of the SC’s visual object representations in the broader context of active vision with saccades. For example, we recently found that SC saccade-related motor bursts are sensory-tuned, and that they still exhibit a preference for real-life object images^33^. This might suggest that peripheral-to-foveal trans-saccadic integration of visual information across eye movements, which can benefit from the SC’s ascending projections to the cortex, may be particularly important for visual object representations.

Besides the short latencies associated with object detection by SC neurons, we were particularly intrigued by the role of spatial frequency information in object detection during the earliest phases (< 100 ms) of SC neural responses. In the early visual burst phase (< 100 ms) of neural responses, we observed that AUC discriminability between the responses for object images and spectral-matched phase-scrambled images was weakened relative to grid scrambling (Fig. 4C,F; but see Fig. 5). This suggests that a functional role for spatial frequency tuning in SC neurons^34^ could be to aid in rapid object detection, especially given that object recognition does indeed make use of spatial frequency information^36–40^. Indeed, this could also mediate rapid orienting responses to objects in periphery^8^, since the spatial frequency tuning of SC neurons is relevant for saccadic reaction times^34^.

Having said that, spatial frequency information cannot fully explain early object detection by the SC because the AUC discrimination performance between intact objects and phase-scrambled controls still became significant earlier than 100 ms after image onset (Figs. 4, 5). In fact, middle spatial frequencies, which are relevant for object recognition^37,38,40^, are associated with slightly delayed SC visual bursts compared to low spatial frequencies^34^. This is consistent with the slight delaying of AUC discrimination performance with phase scrambling relative to grid scrambling. The raw firing rates of the five example neurons of Fig. 3 also all showed higher initial visual burst strengths for real objects relative to phase-scrambled images. These timings are still faster than when some cortical visual areas detect objects^59^. Indeed, early studies have consistently demonstrated that temporal cortex responses to visual objects typically emerge at approximately 100 ms after stimulus onset^60,61^, which is in line with the signal transduction hierarchy along the visual processing stream^62^. All of these observations affirm, in our view, a role for the SC in early detection of visual objects in extrafoveal visual space. This is also consistent with early pop out of high level visual objects, like faces, in perception^7^.

Another interesting observation is that object detection in later intervals after the visual bursts (e.g. > 100 ms after image onset in the phase scrambling results) seems to rely on more than just the spatial frequency information. This is because AUC discrimination was still significant between object and phase-scrambled controls in these later intervals (Fig. 4C,F), and it would imply potential feedback from other visual cortical areas involved in object processing. This could functionally allow visual cortical areas to utilize additional spatial frequency bands, and other rich visual feature representations, beyond those represented in the SC. That is, since SC neurons are predominantly low-pass in nature at our tested eccentricities^34^ (Fig. 2), and since various cortical areas can detect objects at multiple spatial frequency bands^36^, feedback from these areas could help to sustain the object representations in the SC after the initial visual bursts subside. This is important because object recognition does indeed benefit from middle spatial frequencies in images^37,38,40^. It is also important because it demonstrates that at the level of the SC, at least two stages of visual object processing may exist: an early stimulus-induced phase followed by a later more sustained integrative representation.

Related to this, one question that may emerge out of our results is whether SC neurons are indeed preferentially tuned to detect objects, or whether their object responses that we found may reflect instead a tuning to mid-level representations, such as oriented contours. Our grid-scrambled images were meant to maintain local feature representations, but the sizes of our grids may be too small for making an interpretation with regards to longer/larger oriented contours. Thus, our grid scrambling may have disrupted both global object configurations as well as mid-level contour representations. This leaves open the possibility that SC neurons mediate rapid object detection via sensitivity to mid-level feature representations, perhaps like in the second or fourth cortical visual areas^63,64^. However, considering other existing evidence in the literature, we think that it might be unlikely to find strong tuning to mid-level image features in the SC. For example, using reverse correlation techniques, it was found that SC RF’s are generally circularly symmetric, with some neurons showing anisotropies in suppressive surrounds related to the neurons’ locations in the SC topographic map^65^. More importantly, orientation tuning in the SC is relatively weak^9,66,67^. In fact, in our recent comparison of multiple visual features in SC responses, we found that visual response tuning to orientation was the weakest from among the other features that we tested, including spatial frequency, contrast, luminance polarity, and real-life objects^33^. Thus, it remains to be seen whether our results are explained by tuning to mid-level visual representations by SC neurons or not. In our current experiments, we focused on the scrambling controls, to control for low-level features and to be consistent and comparable with the previous studies on object recognition in the cortex, which required many trials in the sessions. Future work utilizing image controls that preserve mid-level features would be necessary to fully answer this question^68^.

Similarly, future work could investigate how object scale relative to the RF size matters. In our experiments, we picked image sizes that fit to our RF sizes and that might be consistent with the statistics of visual object sizes in extrafoveal vision. That is, we were motivated by the idea that extrafoveal objects need to be detected (sometimes rapidly) before they are foveated with eye movements, and that such detection can be mediated by the SC. Thus, we filled the relatively large extrafoveal SC RF’s with objects. However, an equally relevant scenario would be to investigate, perhaps in foveal vision, whether an object that is globally much larger than a given RF size might still be preferentially detected by SC neurons, perhaps through contextual surround effects.

In all, our results motivate further investigations of subcortical pathways for visual perception, particularly given the active nature of behavior in the real world, and the perpetual interplay between sensory processing, on the one hand, and movement generation, on the other. In the same spirit, it would be equally interesting to see whether similar early detection of visual objects as we have found in the SC also appears in the lateral intraparietal area and frontal eye fields, dorsal cortical areas that are classically associated with active orienting behavior.

## Methods

### Experimental animals and ethics approvals

We recorded superior colliculus (SC) neural activity from two adult, male rhesus macaque monkeys (A and M) aged 9 and 8 years, respectively. The experiments were approved by ethics committees at the regional governmental offices of the city of Tübingen (Regierungspräsidium Tübingen), and they were in full accordance with the guidelines and regulations set forth by the German government and the European Union. Our methods are also in accordance with the ARRIVE guidelines.

### Laboratory setup and animal preparation

The experiments were conducted in the same laboratory as that described for the monkey portions of ref.^8^. Briefly, the monkeys were seated in a darkened booth ~ 72 cm from a calibrated and linearized CRT display spanning ~ 31° horizontally and ~ 23° vertically. Data acquisition and stimulus control were managed by a modified version of PLDAPS^69^, interfacing with the Psychophysics Toolbox^70–72^ and an OmniPlex data acquisition system (Plexon, Inc.).

The monkeys were prepared for behavioral training and electrophysiological recordings earlier^73,74^. Specifically, each monkey was implanted with a head-holder and scleral search coil in one eye^73^. The search coil allowed tracking eye movements using the magnetic induction technique^75,76^, and the head-holder comfortably stabilized head position during the experiments. The monkeys also each had a recording chamber centered on the midline and tilted 38° posterior of vertical, allowing access to both the right and left SC.

### Behavioral task

We employed a modified version of the classic delayed, visually-guided saccade task, similar to what we did in our recent behavioral study^8^ (Fig. 1). Each trial started with the appearance of a central white fixation spot of 79.9 cd/m^2^ luminance, presented over a gray background (26.11 cd/m^2^). The fixation spot was 0.18 × 0.18° in dimensions. After 300 ms, an image patch (see below for image preparation procedures) appeared within the visual response fields (RF’s) of the recorded neurons. The image patch could contain pictures of real-life objects or the other versions of image controls described in more detail below, and it had an average luminance of 42.07 cd/m^2^. After 300–700 ms from image patch onset, a white spot identical to the fixation spot appeared on top of a gray disc (diameter: 0.54°; 26.11 cd/m^2^) in the center of the image patch. This white spot was referred to as the saccade target in our analyses. It remained visible (along with the fixation spot and image patch) for 500–1000 ms, at which point the fixation spot disappeared to instruct the monkeys to generate a saccade towards the saccade target (and the underlying image patch). If the monkey successfully made the saccade within 500 ms, it received positive reinforcement in the form of liquid reward.

As described in more detail below, the size of the image patch that we presented was matched to the RF size, and its position was designated after initial assessment of RF locations and sizes. Such assessment was made by running fixation and saccadic RF mapping tasks, and our instantiations of these tasks were described previously^50,74^. Briefly, we performed a visually-guided saccade task with a small white spot (the same size as the fixation spot) as the target. The monkey initially fixated, and an eccentric target appeared. After a random delay, the fixation spot was removed, instructing the monkey to generate a visually-guided rapid eye movement to the target. In the fixation variant of the task, the fixation spot was not removed at trial end, meaning that the monkey did not generate a saccade to the target, and was instead rewarding for maintaining fixation. Target-aligned neural responses in both tasks allowed us to analyze visual responses, and saccade-aligned responses in the saccade version of the task allowed us to assess whether we were in deeper motor-related SC layers or not.

### Image database and image pre-processing procedures

We used a total of 156 grayscale images, from previously published studies^8,31,77^, across seven different object categories: neutral monkey face (15 images), threatening monkey face (15 images), snake (15 images), artificial object (15 images), human hand (16 images), neutral human face (64 images), and fruit (16 images) (Fig. 1; also see Fig. 3A in “Results”). In each session, we randomly picked seven images from the database, one from each category.

For each session, we first sized the images to match the RF sizes of the neurons across the recording contacts. Our neurons spanned eccentricities in the range of 3.1–23.9° (Fig. 2), and we assessed their RF’s using standard visual and saccadic tasks. The image patches were square, and their sizes were in the range of 2–8° (in width and height). These sizes fit within the excitatory parts of the neurons’ RF’s. Since we had multiple RF’s within a session (see neurophysiological procedures below), we picked the image location that best matched most of these RF’s. This was feasible given the topographic organization of the SC and the fact that our electrode penetrations were roughly orthogonal to the SC surface at our recorded eccentricities.

We then iteratively equalized the luminance histograms and spatial frequency spectra of the seven images of a given session using the SHINE toolbox^78^. Specifically, we ran 20 iterations of histogram matching (*histMatch* function) of the gray levels across the images, as well as spectral matching across the same images (*specMatch* function). To generate phase-scrambled images, we randomized the phase matrices of the Fourier-decomposed images, while keeping the amplitude matrices unchanged. Then, to match the real and phase-scrambled images further, we took all object images and their corresponding phase-scrambled images, and we again iteratively matched them once more for histogram levels and frequency spectra using the same SHINE toolbox functions (again, with 20 iterations). Example final images (real and phase-scrambled) are shown in Fig. 1 and also in Fig. 3A of “Results”.

To obtain the grid-scrambled image controls, we overlaid 1-pixel-width horizontal and vertical lines of mean image luminance over the real object images. These horizontal and vertical lines formed a grid of 0.33° × 0.33° squares within which the original object was visible. We then scrambled all grids by randomizing their original locations in the image. To ensure that the neural modulations associated with the grid-scrambled images were not fully explained by the overlaid horizontal and vertical gray lines, we also created the grid overlay without randomizing the individual grid locations. This created the object + grid images (as if the objects were intact and only occluded by a thin grid in front of them). Examples of the final grid-scrambled and object + grid images used in our study are shown in Fig. 1 and in Fig. 3A of “Results”. It should additionally be emphasized here that we were primarily interested in extrafoveal object detection. Thus, the object images occupied extrafoveal SC RF’s, with the individual grids being smaller than the RF’s. This was a deliberate choice that we made in order to disrupt the global configuration of small localized features of the objects within the relatively large extrafoveal RF’s.

### Neurophysiological procedures and functional cell type classification

We recorded neural activity using linear microelectrode arrays (V-Probes, Plexon, Inc.) inserted into the SC. We aligned the arrays (16- or 24-channels with 50 µm inter-electrode spacing) to obtain sufficient coverage across different functional SC layers (0.8–1.2 mm depth coverage by the contacts).

The experiment started by identifying entry into the SC by the deepest electrode contact, and we then advanced the array to insert further contacts into the SC. After ensuring that the tissue had settled and the neural activity was stabilized across contacts, we assessed the RF’s at the electrode contacts using standard visual and saccade tasks. This allowed us to place and size the object images for a given session according to the neurons’ approximate RF locations and sizes. Following RF estimation and the preparation of the object and control images to fit the RF sizes, we ran the main experiment and collected an average of 32 (± 8 SD) trial repetitions per session of the different image conditions that we had: 4 image patch versions (real object, phase-scrambled, grid-scrambled, and object + grid) of each of the 7 object categories (total of 28 different images), resulting in a total of 903 (± 239 SD) trials per session.

We classified neurons as being visual, delay, visual-delay, visual-motor, visual-motor-prelude, or motor in nature, as per previous criteria^79^. Note that such classification is a functional proxy for SC layer information, as known from the literature^80^, and as we also demonstrated from this dataset in a recent study^33^. Specifically, in our delayed visually-guided saccade to image task, we measured the firing rate in each trial, regardless of image conditions, during four different epochs: baseline (100 ms before image onset), visual (50–150 ms after image onset), delay (400–500 ms after saccade target onset), and motor (− 50 to 25 ms from saccade onset). Next, we used the firing rates in these four epochs to compute a non-parametric ANOVA (Kruskal–Wallis), and we determined the neuron class by post-hoc significance tests (p < 0.05). Neurons with significant activity in the visual epoch compared to the baseline epoch were classified as visual neurons. Similarly, neurons with significant activity in the motor epoch compared to the baseline and delay epochs were classified as motor neurons, and a visual neuron with significant motor activity was classified as a visual-motor neuron. Furthermore, visual neurons possessing significant delay-period activity were labeled as visual-delay neurons, and visual-motor neurons with significant delay-period activity were classified as visual-motor-prelude neurons. Any neuron that did not have higher than 5 spikes/s average firing rate in any of the above-mentioned measurement intervals (other than baseline) was excluded from further study. Similarly, for the purposes of this study, we did not analyze the purely motor neurons, since we were interested in assessing visual object detection by the SC.

In total, we had 82 included neurons from monkey A and 131 from monkey M. Approximately half of the neurons in monkey A (47.56%) and two thirds in monkey M (67.18%) were visual-motor-prelude neurons in our database. The next most frequent neuron type in our sample was visual-motor neurons (19.51% in monkey A and 16.03% in monkey M), followed by the motor (13.41% and 6.87%) and visual-delay (13.41% and 5.34%) neurons, and then finally the visual neurons (6.1% and 3.82%). Delay-only neurons were a rarity (1 in monkey M and non-existent in monkey A) and were not analyzed. The neurons’ preferred RF hotspot locations are shown in Fig. 2.

### Data analysis

We detected saccades and microsaccades using our previously described toolbox^81^, and we inspected the detection results manually. To investigate whether microsaccades at image onset might have influenced the SC responses to the stimuli, whether by peri-microsaccadic modulation^45,50^ or jittering of images^47^, we computed microsaccade rate across time from image onset (e.g. Figure 5A,E in “Results”). We did so similarly to how we estimated microsaccade rate recently^8^. Briefly, we binned microsaccades using a 40 ms moving time window, with time steps of 10 ms. In general, we included all trials in our neural data analyses, even when there were microsaccades. This was fine because of the low likelihood of microsaccades, especially in the critical early visual burst interval. However, we also confirmed that our results were unchanged by repeating the analyses after removing all trials in which there was a microsaccade between – 100 and + 300 ms relative to image onset (e.g. Figure 5 in “Results”).

For neural analyses, we sorted the neurons offline using the Kilosort Toolbox^82^, followed by manual curation using the phy software. We then proceeded to analyze the spike rasters and firing rates.

To investigate whether SC visual responses differentiate between object and non-object stimuli, we plotted spike rasters and firing rates across the different image conditions (e.g. Fig. 3 in “Results”). We then assessed whether an ideal observer could discriminate between object and non-object stimuli just based on the SC firing rates. To do so, we performed receiver operating characteristic (ROC) analyses using 40 ms time bins moving in steps of 10 ms. In each 40 ms time bin around the time of image onset, we collected firing rates within this interval from all trials of the real-life object condition and all trials of an image control from the same neuron (e.g. phase-scrambled or grid-scrambled images). We then ran the ROC analysis to obtain an area under ROC curve measure (AUC), allowing us to assess the discriminability between the two firing rate distributions. An area under the ROC curve value of 0.5 would indicate non-discriminable firing rate distributions. We performed the ROC analyses at all times from -100 ms to + 300 ms from image onset, with 10 ms resolution. We did this because the earliest time at which the saccade target could appear in the task was 300 ms (e.g. Fig. 1). We assessed a neuron as detecting objects if its area under the ROC curve in any interval between 0 and 300 ms was statistically significantly different from 0.5. We assessed significance by calculating bootstrapped confidence intervals for the area under the ROC curve measure and using a p < 0.05 criterion. This is similar to our previous approaches^31^. Importantly, we plotted full time courses of AUC values to demonstrate the time-dependent nature of SC neural responses after stimulus onset. We then averaged across all significant neurons’ AUC time courses and obtained 95% confidence intervals across the population. We graphically labeled the time of object detection in figures as the time at which the population AUC discrimination time course first deviated significantly from 0.5 (i.e. no overlap between the 95% confidence interval and 0.5). Of course, this is not meant to be an absolute time of discrimination onset, because it is smoothed by our binning procedure. However, it does still provide an indication of whether object detection is possible in SC neurons within the time frame of their initial visual bursts or later (when feedback from other areas might emerge in the SC neural discharge); this is consistent with the example raw firing rates that we included.

In the ROC analyses described above, we pooled object categories together. Because we ran only one exemplar from each object category in a given session, it was not easy to convincingly assess whether SC neurons also exhibit early object recognition capabilities, besides detecting extrafoveal objects. Future experiments could investigate this possibility in more detail, as in, for example, the studies investigating SC face preference^15–19^.

We also repeated the ROC analyses for the different functionally-classified neurons. For example, we picked only visual-motor-prelude neurons and calculated the area under the ROC curve metrics for those, or we only considered visual-delay neurons. This allowed us to assess whether early visual object detection by the SC (e.g. in the initial visual burst interval; see “Results”) only occurred in purely sensory neurons, or whether it also appeared in deeper visual-motor neurons. In some analyses, we found that whether a neuron had delay-period activity or not (e.g. visual-delay and visual-motor-prelude neurons both had delay-period activity) influenced the ROC results in either early or late intervals after image onset. Therefore, to demonstrate this point, we combined neuron types appropriately; that is, visual-motor-prelude and visual-delay neurons were combined since they both showed delay-period activity, and visual-motor or visual neurons were combined together because they both lacked delay-period activity.

In all figures and analyses, we showed results for each monkey individually.

### Experimental design and statistical analyses

We minimized sampling bias by recording neurons using linear micro-electrode arrays and later sorting them offline. We also replicated results in each monkey separately, and we had sufficient sampled neurons to obtain a rough estimate of proportions of neurons showing the effects that we were analyzing. For each neuron, we also ensured collecting > 30 repetitions per condition, allowing for robust within-neuron analyses.

We provided descriptive statistics in all figures, showing numbers of replicates and variability measures. We also showed individual monkey results separately.

All statistical tests were described above and are reported in “Results”.

## Data Availability

The datasets generated during and/or analysed during the current study are available from the corresponding author on reasonable request.
